# Chitosonic^®^ Acid as a Novel Cosmetic Ingredient: Evaluation of its Antimicrobial, Antioxidant and Hydration Activities

**DOI:** 10.3390/ma6041391

**Published:** 2013-03-28

**Authors:** Shu-Mei Lee, Kun-Ho Liu, Yen-Yu Liu, Yen-Po Chang, Chih-Chien Lin, Yi-Shyan Chen

**Affiliations:** 1 Department of Cosmetic Science and Management, Mackay Medicine, Nursing and Management College, 92 Shengjing Road, Beitou, Taipei 11260, Taiwan; E-Mail: s107@eip.mkc.edu.tw; 2 Advanced Delivery Technology Co. Ltd, 5F, D Building, No.120, Zhonghua Rd., Hsinchu Industrial Park, Hukou Township, Hsinchu 30352, Taiwan; E-Mails: anobbona@gmail.com (K.-H.L.); yyliu0527@gmail.com (Y.-Y.L.); ypchang1021@gmail.com (Y.-P.C.); 3 Department of Cosmetic Science, Providence University, No. 200, Sec. 7, Taiwan Boulevard, Shalu, Taichung 43301, Taiwan

**Keywords:** antimicrobial activity, antioxidant activity, Carboxymethyl Caprooyl Chitosan, Chitosonic^®^ Acid, cosmetic ingredient, hydration activity

## Abstract

Chitosonic^®^ Acid, carboxymethyl hexanoyl chitosan, is a novel chitosan material that has recently been accepted by the Personal Care Products Council as a new cosmetic ingredient with the INCI (International Nomenclature of Cosmetic Ingredients) name Carboxymethyl Caprooyl Chitosan. In this study, we analyze several important cosmetic characteristics of Chitosonic^®^ Acid. Our results demonstrate that Chitosonic^®^ Acid is a water-soluble chitosan derivative with a high HLB value. Chitosonic^®^ Acid can form a nano-network structure when its concentration is higher than 0.5% and can self-assemble into a nanosphere structure when its concentration is lower than 0.2%. Chitosonic^®^ Acid has potent antimicrobial activities against gram-positive bacteria, gram-negative bacteria and fungus. Chitosonic^®^ Acid also has moderate DPPH radical scavenging activity. Additionally, Chitosonic^®^ Acid exhibits good hydration activity for absorbing and retaining water molecules with its hydrophilic groups. From a safety point of view, Chitosonic^®^ Acid has no cytotoxicity to L-929 cells if its concentration is less than 0.5%. Moreover, Chitosonic^®^ Acid has good compatibilities with various normal cosmetic ingredients. Therefore, we propose that Chitosonic^®^ Acid has the potential to be a widely used ingredient in various types of cosmetic products.

## 1. Introduction

In recent years, the cosmetic industry has been a rapidly growing industry worldwide. The continuous development of new active ingredients for cosmetics and personal care products is one of the most important areas of research in this industry [1,2]. As a result, there are a significant number of novel cosmetic products that are based on this new generation of active ingredients [3].

Chitosan is the common name of the linear, random copolymer that consists of β-(1-4)-linked D-glucosamine and *N*-acetyl-D-glucosamine. The molecular structure of chitosan consists of a linear backbone linked with glycosidic bonds [4,5]. Chitosan is the major component of crustacean shells such as crab, shrimp, krill and crawfish shells. Additionally, chitosan is the second most abundant natural biopolymer after cellulose [6]. Commercial chitosan samples are typically prepared by chemical de-*N*-acetylation of chitin under alkaline conditions. Depending on the source of the natural chitin (extracted from shells) and its production process, chitosan can differ in size (average molecular weight *M*_w_) and degree of *N*-acetylation (DA) [7,8]. The poor solubility of chitosan in water and in common organic solvents has restricted its application. However, reactive amino groups in the chitosan backbone make it possible to chemically conjugate chitosan with various biological molecules and to improve its utilization [4,9,10]. The modified chitosan molecules exhibit various potential biological activities, such as antimicrobial, antifungal, antitumor and immunostimulatory activities. Moreover, modified chitosan molecules are also used in drug/gene delivery, biosensors, wound healing and tissue regeneration and as carriers of immobilized enzymes and cells [11,12,13,14].

**Figure 1:**
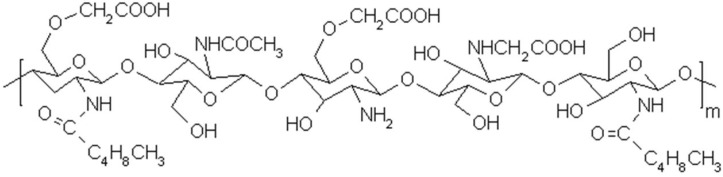
Molecular structure of Chitosonic^®^ Acid, carboxymethyl hexanoyl chitosan.

Chitosonic^®^ Acid, carboxymethyl hexanoyl chitosan (Figure 1), is a novel chitosan material that has recently been accepted by the Personal Care Products Council as a new cosmetic ingredient with the INCI (International Nomenclature of Cosmetic Ingredients) name Carboxymethyl Caprooyl Chitosan. Although some articles have reported that chitosan and its derivatives can be used as a delivery system for cosmetics [15,16,17,18], to the best of our knowledge, no studies have demonstrated that chitosan and its derivatives can be used as key active ingredients in the formulation of cosmetics. Therefore, in this study, we analyze several important characteristics and activities of Chitosonic^®^ Acid, including the average molecular weight, solubility in water, zeta-potential, isoelectric point, hydrophilic-lipophilic balance (HLB) value and antimicrobial, antioxidant and hydration activities. Moreover, the cytotoxicity of Chitosonic^®^ Acid to mouse fibroblast L-929 cells has been studied to determine its safety. From these results, we propose that this chitosan derivative may be used as an active ingredient in cosmetic products.

## 2. Results and Discussion

Chitosonic^®^ Acid (carboxymethyl hexanoyl chitosan) was synthesized according to the procedure used in our earlier studies, and its structure is shown in Figure 1. The synthesis procedures of Chitosonic^®^ Acid are listed in the Experimental Section. In the present study, we first analyzed several important characteristics of Chitosonic^®^ Acid. Then, the antimicrobial, antioxidant and hydration activities of Chitosonic^®^ Acid were examined. Finally, the safety of Chitosonic^®^ Acid was evaluated using mouse fibroblast L-929 cells via a cytotoxicity assay.

### 2.1. Characteristics of Chitosonic^®^ Acid

#### 2.1.1. Molecular Weight and Solubility of Chitosonic^®^ Acid

As shown in Table 1, the average molecular weight of Chitosonic^®^ Acid is approximately 160,000 g/mol. This value indicates that Chitosonic^®^ Acid is not a high molecular weight chitosan derivative. The molecular weight of the chitosan used to prepare Chitosonic^®^ Acid is 215,000 g/mol. Thus, the produced chitosan derivative has a lower molecular weight. This result is mainly due to the powerful and long-term agitation procedures of Chitosonic^®^ Acid. These processes can reduce the molecular weight of Chitosonic^®^ Acid via a strong shear stress. Moreover, the molecular weight is also changed by the substitution degrees of the carboxymethyl (DC) and hexanoyl (DH) groups. In our earlier study, this chitosan derivative was analyzed to determine the substitution degrees of the carboxymethyl and hexanoyl groups, which are a DC of 0.24 and a DH of 0.33 [19]. The DC and DH affect the solubility of chitosan in water. The water solubility of Chitosonic^®^ Acid is approximately 5% (Table 1) and thus can be broadly used in aqueous formulations. Additionally, from a biological point of view, relatively low molecular weight chitosan derivatives may have good biodegradability, antimicrobial activity and antioxidant activity [13].

**Table 1:** Some important characteristics of Chitosonic^®^ Acid.

Characteristic	Value
Average molecular weight	~160,000 g/mol
Solubility in water	~5%
Zeta-potential	29 mV (pH 5)
Isoelectric point	pH 4.6
HLB value ^a^	36.5

Note: ^a^ HLB: hydrophilic-lipophilic balance.

#### 2.1.2. Zeta potential, Isoelectric Point and HLB Value of Chitosonic^®^ Acid

The zeta potential of a particle represents the charge on the particle surface. The zeta potential of Chitosonic^®^ Acid (0.1%) in a pH 5 aqueous solution was determined to be 29 mV. The isoelectric point of Chitosonic^®^ Acid is pH 4.6 (Table 1). The substitution degree of these functional groups may change the zeta potential of the chitosan derivatives. In our earlier study, if the DC of the chitosan derivatives was increased to 0.5, the zeta potential of the chitosan derivatives decreased to a negative value [4]. This result was due to a higher carboxymethyl group substitution, which led to a higher negative charge captured on the particle surface. Therefore, for a low pH value, the surface of Chitosonic^®^ Acid (DC of 0.25) is occupied by a positive charge.

The hydrophilic-lipophilic balance (HLB) value is one of the most important properties of an amphiphilic molecule. The HLB value can be used to determine if the molecule is hydrophilic or lipophilic and to predict its usage. For Chitosonic^®^ Acid, the HLB value, shown in Table 1, is 36.5. This result demonstrated that Chitosonic^®^ Acid is a more hydrophilic molecule. Furthermore, Chitosonic^®^ Acid can be used as an emulsifier in oil-in-water emulsions.

#### 2.1.3. Morphological Evaluation of Chitosonic^®^ Acid

**Figure 2:**
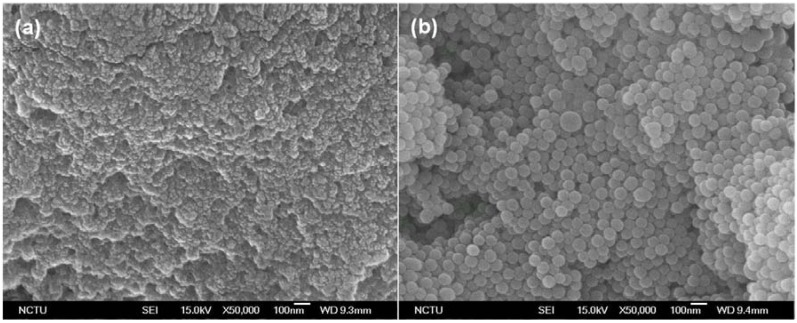
(**a**) Scanning electron microscopy (SEM) image of Chitosonic^®^ Acid at a high concentration (0.5%); (**b**) SEM image of Chitosonic^®^ Acid at a low concentration (0.2%).

A morphological evaluation of Chitosonic^®^ Acid was conducted using scanning electron microscopy (SEM), and the results are shown in Figure 2. For Chitosonic^®^ Acid at a relatively high concentration (0.5%), this chitosan derivative formed a thin film with a nano-network structure (Figure 2a). However, if the concentration of Chitosonic^®^ Acid was decreased to 0.2%, it has a perfect spherical geometry ranging in size from 50 to 100 nm in diameters (Figure 2b). This phenomenon might be caused by the high-concentration Chitosonic^®^ Acid forming chitosan chains that interact with each other. Therefore, the nano-network structure is exhibited if the concentration of Chitosonic^®^ Acid is higher than 0.5%. In contrast, if the Chitosonic^®^ Acid concentration was reduced to 0.2%, the amphiphilic characteristic of Chitosonic^®^ Acid was dominant, and self-assembled nanocapsules formed. This feature of Chitosonic^®^ Acid is also the reason for we used a relative low concentration of Chitosonic^®^ Acid (0.1%) to examine its zeta potential value.

In formulations, Chitosonic^®^ Acid can be used as a good moisturizing agent to capture water molecules using the nano-network structure when its concentration is at least 0.5%. If the concentration of Chitosonic^®^ Acid is lower than 0.2%, it can also be used as a carrier to deliver and/or protect active ingredients using the self-assembled nanospheres. Therefore, we propose that Chitosonic^®^ Acid has the potential to be used as an ingredient in moisture and is suitable as the carrier of a delivery system.

### 2.2. Antimicrobial and Antioxidant Activities of Chitosonic^®^ Acid

#### 2.2.1. Antimicrobial Activity of Chitosonic^®^ Acid

The antimicrobial activities of Chitosonic^®^ Acid are shown in Table 2. These results demonstrated that all types of microorganisms can clearly be suppressed by 2% Chitosonic^®^ Acid after an 18 h treatment. The inhibition rates range from 87.3% up to over 99%. Gram-negative bacteria were more susceptive to Chitosonic^®^ Acid than other microorganisms. When treated with *Staphylococcus epidermidis*, Chitosonic^®^ Acid had the lowest antimicrobial activity (Table 2). The antimicrobial activities of chitosan and its derivatives are well described in many previous studies. The mechanism of the chitosan antimicrobial activity has not been fully clarified. However, the most likely mechanism is a change in cell permeability due to the interactions between the positively charged chitosan molecules and the negatively charged microbial cell membranes. This interaction leads to the leakage of proteinaceous and other cellular components [6,7,20,21]. Chitosonic^®^ Acid is a chitosan derivative with a 160,000 g/mol average molecular weight. When it is applied on skin, we suppose that most Chitosonic^®^ Acid molecules will retain on the surface. Thus, we propose that Chitosonic^®^ Acid can also be used as an antimicrobial agent in formulations at higher concentrations.

**Table 2:** Antimicrobial and antioxidant activities of Chitosonic^®^ Acid.

Antimicrobial activity		
Microorganism ^a^	Type	Inhibition rate (%)
*Escherichia coli*	gram-negative bacterium	>99.99%
*Pseudomonas aeruginosa*	gram-negative bacterium	>99.99%
*Propionibacterium acnes*	gram-positive bacterium	99.9%
*Staphylococcus epidermidis*	gram-positive bacterium	87.3%
*Staphylococcus aureus*	gram-positive bacterium	95.6%
*Methicillin-resistant Staphylococcus aureus*	gram-positive bacterium	91.4%
*Candida albicans*	diploid fungus	99.4%
**Antioxidant activity**		
**Ingredient**	**Free radical ^b^**	**Scavenging rate (%)**
1% Arbutin	DPPH	84%
1% Hyaluronic acid	DPPH	0%
1% Chitosonic^®^ Acid	DPPH	66%
4% Chitosonic^®^ Acid	DPPH	89%

Note: ^a^ For antimicrobial activity, all groups were tested using 2% CA for 18 h; ^b^ DPPH, 2,2-diphenyl-1-picrylhydrazyl.

#### 2.2.2. Antioxidant Activity of Chitosonic^®^ Acid

The antioxidant activity of Chitosonic^®^ Acid was tested using the scavenging effect on 2,2-diphenyl-1-picrylhydrazyl (DPPH) radicals. The results are shown in Table 2. The scavenging rates of 1% and 4% Chitosonic^®^ Acid were 66% and 89%, respectively. Compared with the frequently used antityrosinase and antioxidant agent arbutin [22], Chitosonic^®^ Acid has moderate to good antioxidant activity for scavenging DPPH radicals. The antioxidant activities of chitosan and its derivatives were described in many earlier studies [6,21,23,24]. We believe that this property of Chitosonic^®^ Acid can be useful for its application in various cosmetic products.

### 2.3. Hydration Activity of Chitosonic^®^ Acid

**Figure 3:**
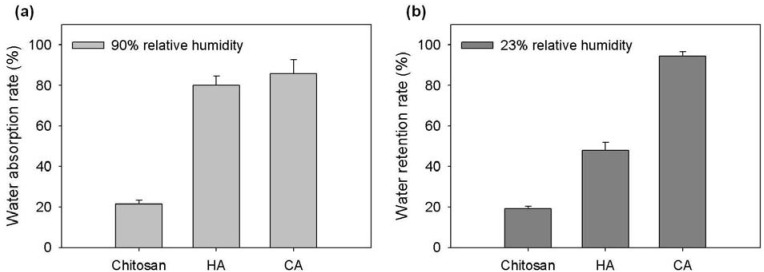
(**a**) Water absorption rate of Chitosonic^®^ Acid in 90% relative humidity; (**b**) Water retention rate of Chitosonic^®^ Acid in 23% relative humidity. HA: hyaluronic acid, CA: Chitosonic^®^ Acid.

To further examine the hydration activity of Chitosonic^®^ Acid, we analyzed its water absorption and retention abilities in different relative humidities. The results are shown in Figure 3. In 90% relative humidity, 0.2 g of Chitosonic^®^ Acid absorbed over 85% of the water within 48 h (Figure 3a). The water absorption ability of Chitosonic^®^ Acid is clearly higher than that of chitosan, which has a similar value to that of hyaluronic acid. From a study on the water retention ability, 2% Chitosonic^®^ Acid retains approximately 93% of the absorbed water in 23% relative humidity over a period of 24 h (Figure 3b). The water retention ability of Chitosonic^®^ Acid is clearly higher than those of chitosan and hyaluronic acid. This result indicates that higher concentrations of Chitosonic^®^ Acid (higher than 0.5%) can exhibit good hydration activity for retaining water molecules in its nano-network structure (Figure 2a and Figure 3b).

**Figure 4:**
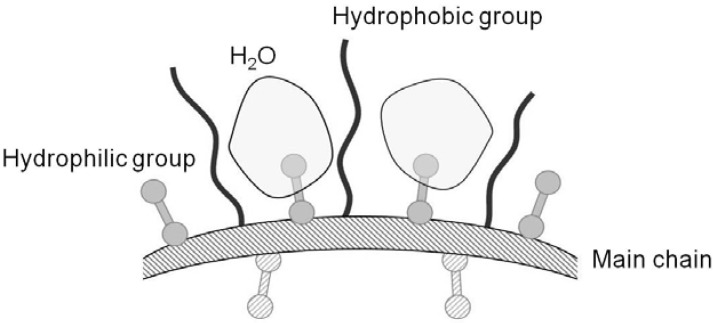
Illustration of the interaction between water and Chitosonic^®^ Acid molecules.

The molecular structure of Chitosonic^®^ Acid is a linear backbone with carboxymethyl and hexanoyl side chains (Figure 1). Therefore, the hydrophilic and hydrophobic properties of Chitosonic^®^ Acid are due to the carboxymethyl and hexanoyl groups, respectively. An illustration of the interaction between water and Chitosonic^®^ Acid is shown in Figure 4. The water molecules combine with the Chitosonic^®^ Acid hydrophilic groups between the hydrophobic side chains (Figure 4). We believe that the nano-network structure of higher concentration Chitosonic^®^ Acid may attract water into its molecule. This feature leads to excellent hydration activity for Chitosonic^®^ Acid.

### 2.4. Cytotoxicity of Chitosonic^®^ Acid in Mouse Fibroblast L-929 Cells

**Figure 5:**
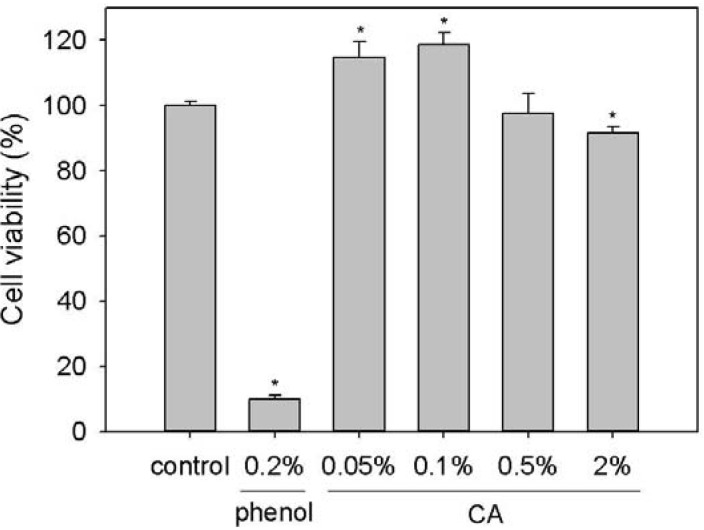
Cell viability of L-929 cells treated with various concentrations of Chitosonic^®^ Acid. Each value is expressed as the mean ± S.E. (*n* = 3). *P < 0.05 compared with the control. CA: Chitosonic^®^ Acid.

To confirm the cytotoxicity of Chitosonic^®^ Acid, we used mouse fibroblast L-929 cells as a model to analyze the viability of Chitosonic^®^-Acid-treated cells using a standard 3-(4,5-Dimethylthiazol-2-yl)-2,5-diphenyl tetrazolium bromide (MTT) assay. As shown in Figure 5, 0.2% phenol exhibited a large cytotoxicity to L-929 cells. The cell viability of Chitosonic^®^-Acid-treated L-929 cells exhibited no cytotoxicity if the concentration is lower than 0.5%. When concentration of Chitosonic^®^ Acid is higher than 2%, the cell viability will slightly decreased to approximate 92%. This result might be caused by that Chitosonic^®^ Acid will form a nano-network structure at high concentrations (higher than 0.5%) and then directly contact/cover the tested cells. Additionally, 0.05% and 0.1% Chitosonic^®^ Acid enhanced the growth of L-929 cells up to approximately 16% and 20%, respectively (Figure 5). These results support the fact that Chitosonic^®^ Acid is a safe ingredient and can be incorporated into cosmetic products.

To confirm the ability of Chitosonic^®^ Acid to be used in cosmetic formulations, we tested the compatibility of Chitosonic^®^ Acid with some frequently used cosmetic ingredients. The results demonstrated that Chitosonic^®^ Acid has good compatibilities with polymers, such as methyl cellulose and hydroxypropyl cellulose, emulsifiers, such as sorbitan monolaurate, sorbitan monooleate and polyoxyethylene lauryl ether and several moisturizing components, such as glycerin, 1,3-butylene, ceramide and collagen (data not show). Moreover, Chitosonic^®^ Acid can be incorporated with many functional ingredients, such as skin whitening agents, anti-aging agents, botanical extracts and essential oils (data not show). These results also indicated that Chitosonic^®^ Acid has the potential to be widely used as a cosmetic ingredient.

## 3. Experimental Section

### 3.1. Materials

Chitosan (*M*_w_ = 215,000 g/mol, deacetylation degree = 85%–90%) was supplied from Sigma-Aldrich (St. Louis, MO, USA). Sodium hydroxide, 2-Propanol, chloroacetic acid, hexanoyl anhydride, sodium acetate, 2,2-diphenyl-1-picrylhydrazyl (DPPH), phenol and hyaluronic acid (*M*_w_ = 2,000,000 g/mol) were also purchased from Sigma-Aldrich. Arbutin was purchased from Alfa Aesar (Ward Hill, MA, USA). Modified Eagle’s medium (MEM) and fetal bovine serum (FBS) were purchased from Gibco BRL/Invitrogen (Carlsbad, CA, USA). MTT, 3-(4,5-Dimethylthiazol-2-yl)-2,5-diphenyl tetrazolium bromide, was purchased from Affymetrix/USB (Cleveland, OH, USA). Deionized distilled water (ddH_2_O), used in solutions, was obtained from a Milli-Q system (Millipore, Bedford, MA, USA).

### 3.2. Synthesis of Chitosonic^®^ Acid

Following the procedure in our previous study, chitosan (5 g) was suspended in 50 mL of 2-propanol at room temperature and dissolved for 30 min. The suspension was gently mixed with 12.5 mL of a NaOH solution. The mixture (containing 13.3 M NaOH) was then mixed with chloroacetic acid to prepare a carboxymethyl chitosan sample with a low degree of carboxymethyl substitution. The obtained and dried sample (2 g) was dissolved in ddH_2_O (50 mL) under stirring for 24 h. The resulting solution was mixed with methanol (50 mL), followed by the addition of 0.5 M hexanoyl anhydride to generate a hexanoyl substitution. After a reaction time of 12 h, the Chitosonic^®^ Acid solutions were collected using a dialysis membrane (Sigma-Aldrich) after dialysis against an ethanol solution (25% v/v) for 24 h [4].

### 3.3. Characterization

#### 3.3.1. Molecular Weight and Solubility

The average molecular weight was determined using a viscometric method [25]. Chitosonic^®^ Acid samples were prepared in 0.2 M acetic acid with 0.1 M sodium acetate solutions. The relative viscosity of the Chitosonic^®^ Acid samples and the solvent were measured using an Ubbelohde capillary viscometer at 25 °C. The average molecular weight was then calculated based on the Mark-Houwink equation [25].

The solubility was determined by dissolving 1 g of Chitosonic^®^ Acid in 10 g of ddH_2_O (pH 7) under moderate stirring for 12 h at 90 °C. The solution was then centrifuged. The supernatant and precipitate were separated by decanting. Finally, the supernatant was lyophilized and weighed. The solubility of Chitosonic^®^ Acid in water was expressed as a percentage of the solution [26].

#### 3.3.2. Zeta-potential, Isoelectric Point and Hydrophilic-Lipophilic Balance (HLB) Value

The zeta potential measurement of Chitosonic^®^ Acid (0.1%) in an aqueous suspension was determined using a Zetasizer 169 HS3000 photon correlation spectrometer (Malvern Instruments, UK). The zeta potentials of Chitosonic^®^ Acid at various pH conditions were analyzed and then used to calculate the isoelectric point. The isoelectric point is the pH value where the zeta potential is zero.

The hydrophilic-lipophilic balance (HLB) value was analyzed following the procedure of an earlier study with some modifications [27]. A standard emulsifier, Span 80 (HLB = 4.3), was mixed with different proportions of Chitosonic^®^ Acid. The mixed emulsifier was then combining with water and silicone oil (required HLB = 10.5 for O/W emulsion) in a 1:89:10 (w/w/w) ratio. The HLB of the most stable Chitosonic^®^-Acid-containing emulsion was equal to the required HLB of silicone oil. The HLB value of Chitosonic^®^ Acid was then calculated using the required HLB.

#### 3.3.3. Morphological Evaluation by Scanning Electron Microscopy (SEM)

Morphological evaluations of Chitosonic^®^ Acid at different concentrations were performed using scanning electron microscopy (SEM) (S6500, JEOL, Japan). For SEM observations, the samples were suspended in anhydrous ethanol and then dip-coated onto a silicon substrate. After evaporation at 50 °C for 24 h, the dried samples were coated with gold 20 nm thick for analysis.

### 3.4. Antimicrobial Assay

The antimicrobial effects of Chitosonic^®^ Acid were tested using several microorganisms (obtained from the Bioresource Collection and Research Center, BCRC, Hsinchu, Taiwan), such as *Escherichia coli* (BCRC 10675) and *Pseudomonas aeruginosa* (BCRC 10944), which were selected as examples of gram-negative bacteria, *Propionibacterium acnes* (BCRC 10723), *Staphylococcus epidermidis* (BCRC 10783), *Staphylococcus aureus* (BCRC 10451) and methicillin-resistant *Staphylococcus aureus* (MRSA), which were selected as examples of gram-positive bacteria, and *Candida albicans* (BCRC 20511), which was selected as an example of a diploid fungus. To test the antimicrobial activity of Chitosonic^®^ Acid, a 2% concentration of Chitosonic^®^ Acid was used to treat all of the microbials for 18 h, and then a modified Japanese Industrial Standard JIS Z 2801:2000 [28] procedure was performed. Briefly, all microbials were cultured in their own standard culture medium. In the period of analysis, 0.1 mL of tested Chitosonic^®^ Acid was mixed and inoculated with 0.4 mL of each early-stationary phase microorganism culture containing 10^5^ to 10^6^ CFU/mL at 37 °C (excluding *C. albicans*, which is cultured in 23 °C) for 18 h. After inoculation, the tested samples were gently homogenized and then the serial dilutions were carried out to verify the colonies after 72 h incubation at 23 °C for *C. albicans* or 24 h incubation at 37 °C for other microbials.

### 3.5. Antioxidant Assay

To determine the scavenging effect on 2,2-diphenyl-1-picrylhydrazyl (DPPH) radicals, 0.1 mL of each concentration of arbutin, hyaluronic acid and Chitosonic^®^ Acid were diluted with 0.4 mL of a Tris-HCl buffer (100 mM, pH 7.4) and then individually mixed with 0.5 mL of a methanolic solution containing DPPH radicals. The final concentration of DPPH was 0.25 mM. The mixture was shaken vigorously and left to stand for 20 min at 25 °C in the dark. The absorbance was then determined at 517 nm [29].

### 3.6. Hydration Assay

To characterize the water absorption ability of Chitosonic^®^ Acid, 0.2 g of the tested samples, including chitosan, hyaluronic acid and Chitosonic^®^ Acid, were placed in an incubator with a relative humidity of 90% at 25 °C for 48 h. The water absorption rate was determined using the weight change percentage of the dry sample. The water retention ability was evaluated using the water mobility during deswelling. Fully swollen hydrogels, including 2% chitosan, hyaluronic acid and Chitosonic^®^ Acid, were placed in an incubator with a relative humidity of 23% at 25 °C for 24 h. The water retention rate was calculated using the weight alteration percentage of the wet sample [19].

### 3.7. Cytotoxicity Assay

L-929 cells (NCTC clone 929, mouse fibroblast, BCRC 60091) were seeded in 96-well plates (1 × 10^4^ cells/well) using an MEM medium supplemented with 10% FBS for 24 h. The prepared cells were subsequently treated with different concentrations of the samples (0.05% to 0.5%) for 48 h. Next, 100 μL (0.5 mg/mL) of the MTT solution was added to the cells, which were then incubated at 37 °C for 30 min and washed twice with PBS. Finally, the PBS-cleaned cells were lysed with 100 μL of DMSO, and the absorbance was measured spectrophotometrically at 570 nm using an ELISA reader [30].

### 3.8. Statistical Analysis

All analytic measurements were performed in triplicate. The results were analyzed using Student’s *t*-test and were expressed as the mean ± standard deviation for each measurement. *P*-values less than 0.05 were considered to be significant.

## 4. Conclusions

In summary, the novel cosmetic ingredient Chitosonic^®^ Acid is a water-soluble chitosan derivative with a high HLB value. Chitosonic^®^ Acid can form a nano-network structure when its concentration is higher than 0.5% and can self-assemble into a nanosphere structure when its concentration is lower than 0.2%. Chitosonic^®^ Acid has potent antimicrobial activities against gram-positive bacteria, gram-negative bacteria and fungus. Chitosonic^®^ Acid also has moderate DPPH radical scavenging activity. Additionally, Chitosonic^®^ Acid exhibits good hydration activity for absorbing and retaining water molecules. From a safety point of view, Chitosonic^®^ Acid has no cytotoxicity to L-929 cells if its concentration is less than 0.5%. Moreover, Chitosonic^®^ Acid has good compatibility with many ingredients that are commonly used in cosmetics. Therefore, we propose that Chitosonic^®^ Acid has the potential to be a widely used ingredient in various types of cosmetic products.
